# Supplementary material for: Genetic mapping of etiologic brain cell types for obesity
Source: eLife. 2020 Sep 21;9:e55851. doi: 10.7554/eLife.55851 (PMC7505664; doi:10.7554/eLife.55851)
Supplement: Appendix 2—figure 1—source data 1. [file elife-55851-app2-fig1-data1.docx]

| **ES metric abbr.** | **ES metric name** | ***ES_w_* scale** | ***ES_w_* interpretation** | **Reference** |
| --- | --- | --- | --- | --- |
| GES | Gene Enrichment Score | $\mathbb{R}_{\geq0}$ | <1: *g* depleted in *c*  1: *g* no enrichment  >1: *g* enriched in *c* | Zeisel (*Cell*, 2018) |
| EP | Expression Proportion | [0, 1] | 0: *g* not expressed in *c*  0.5: *c* makes up 50% of *g* total mean expression.  1: *g* uniquely expressed in *c* | Skene (*Nature Genetics*, 2018) |
| NSI | Normalized Specificity Index | [0, 1] | 0: *g* not expressed in *c*  0.5: *g*’s mean expression fold-change (focal cell type compared to other cell types) is on average within the top 50% of all genes  1: *g*’s mean expression fold-change is the largest fold-change observed over all genes. | Modified from Dougherty (*Bioinformatics*, 2010) |
| DET | Differential Expression T-statistic | $\mathbb{R}$ | <0: *g’s* mean expression lower in *c*  >0: *g’s* mean expression higher in *c* | - |
